# Combined Papillectomy and Autologous Conjunctival Membrane Graft as Management of Giant Papillae for Severe, Refractory Palpebral Vernal Keratoconjunctivitis—A Case Report

**DOI:** 10.1155/2024/9973441

**Published:** 2024-08-05

**Authors:** Devina Nur Annisa, Hernawita Suharko, Hasiana Lumban Gaol, Viona Viona

**Affiliations:** ^1^ Pediatric Ophthalmology and Strabismus JEC Eye Hospitals and Clinics, Jakarta, Indonesia; ^2^ Orbital, Oculoplastic, and Reconstructive JEC Eye Hospitals and Clinics, Jakarta, Indonesia; ^3^ Department of Research JEC Eye Hospitals and Clinics, Jakarta, Indonesia

## Abstract

**Introduction:** Vernal keratoconjunctivitis (VKC) is an allergic disease that predominantly affects young individuals, with a higher incidence among males. Traditionally seen as a condition of childhood that resolves at puberty, recent studies have shown persistent cases in some individuals, potentially influenced by hot and dry tropical environmental conditions. VKC is more prevalent in regions with a hot and humid climate and a high presence of airborne allergens, leading to significant morbidity and impacting the quality of life for affected individuals. Severe and chronic cases can lead to vision-threatening corneal complications, making effective management crucial. Although the clinical profile of VKC varies geographically, comprehensive studies in specific regions remain limited.

**Case Presentation:** In this case report, we present an 18-year-old male patient with severe and refractory VKC. Despite initial treatments, including topical and oral medications, recurrent episodes occurred every 6 months, accompanied by giant papillae (GP) formation and corneal ulcers. The patient had a history of triamcinolone injection and papillectomy combined with amnion membrane transplantation, but presented with a refractory disease in a year. Surgical intervention involving papillectomy and autologous conjunctival membrane graft was performed, leading to a smooth upper tarsal conjunctiva during the 2-year follow-up period, without GP recurrence and maintaining a clear cornea. The patient's symptoms were effectively managed with topical medications.

**Conclusion:** The management of VKC necessitates a comprehensive approach involving preventive measures, pharmacological treatment, and surgical interventions for refractory cases. This case highlights the potential benefits of surgical techniques, such as papillectomy and autologous conjunctival membrane graft, in managing severe and refractory VKC cases with a history of previous surgical procedure failure. However, it is essential to recognize that exposure avoidance and allergic control remain fundamental in VKC management. Further research and larger studies are required to validate the efficacy of these surgical techniques in managing VKC.

## 1. Introduction

Vernal keratoconjunctivitis (VKC) is an allergic eye disease primarily affecting young individuals, with a higher prevalence in males [1, 2]. Traditionally considered a childhood condition that resolves during puberty, a study in India reported 12% of patients being over 20 years old, suggesting prolonged VKC in some cases, potentially due to hot and dry tropical environments [3]. VKC exhibits higher prevalence in hot and humid regions with significant airborne allergens, resulting in substantial morbidity and impaired quality of life [4, 5]. Severe and chronic cases can lead to vision-threatening corneal complications, while treatments pose additional concerns for managing this ocular surface disorder [6, 7, 8].

VKC is a bilateral allergic inflammation of the conjunctiva, characterized by recurrent seasonal episodes and gelatinous hypertrophy in the limbal region and/or large conjunctival papillae in the upper tarsal region. Although relatively rare in temperate regions, it represents a significant cause of hospital referrals in parts of Africa and Asia. Investigations have revealed VKC's complex immunopathogenesis, involving both IgE-dependent (Type I allergic) and IgE-independent (Type IV allergic) mechanisms, with various inflammatory cells contributing to the release of chemical mediators [1, 2, 4].

Clinically, VKC is distinguished by proliferative alterations in the tarsal or limbal conjunctiva, appearing as papillary hyperplasia (palpebral form), gelatinous swelling or hypertrophy (limbal form), or a combination (mixed form) [6]. Corneal lesions may also be observed, further complicating the condition. Pharmacological treatment, including antiallergic and steroid eye drops, is the primary approach for managing VKC and other allergic conjunctival diseases, with additional interventions considered for severe cases [9, 10].

## 2. Case Presentation

An 18-year-old male patient initially presented to our clinic at the age of 11 with recurring complaints of itchiness and excessive tearing, along with a history of corneal ulcer in the left eye. During the first visit in 2015, papillae were identified on the superior tarsal conjunctiva of both eyes. The patient was diagnosed with vernal conjunctivitis and received topical and oral treatments, including gatifloxacin eye drops, prednisolone acetate eye drops, fusidic acid eye ointment, and a combination of oral antihistamines and steroids. However, 6 months later, the patient reported experiencing the same symptoms, and papillae and ropey discharge were observed in both eyes. Despite receiving extensive management for vernal conjunctivitis, it can be concluded that episodes occur approximately twice a year, or every 6 months.

**Figure 1:**
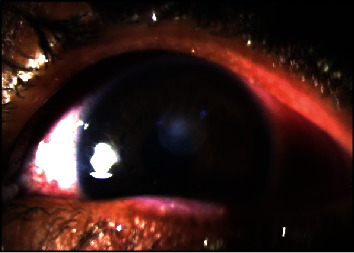
Shield ulcer on the left eye.

**Figure 2:**
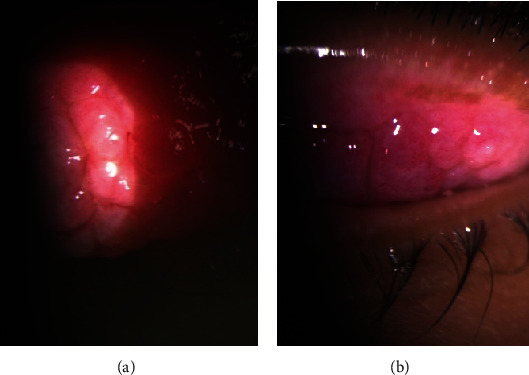
(a) Right eye. (b) Left eye.

**Figure 3:**
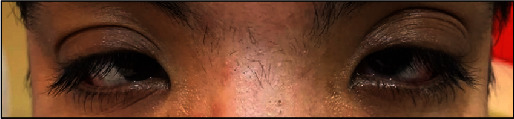
Mechanical ptosis on both eyes.

**Figure 4:**
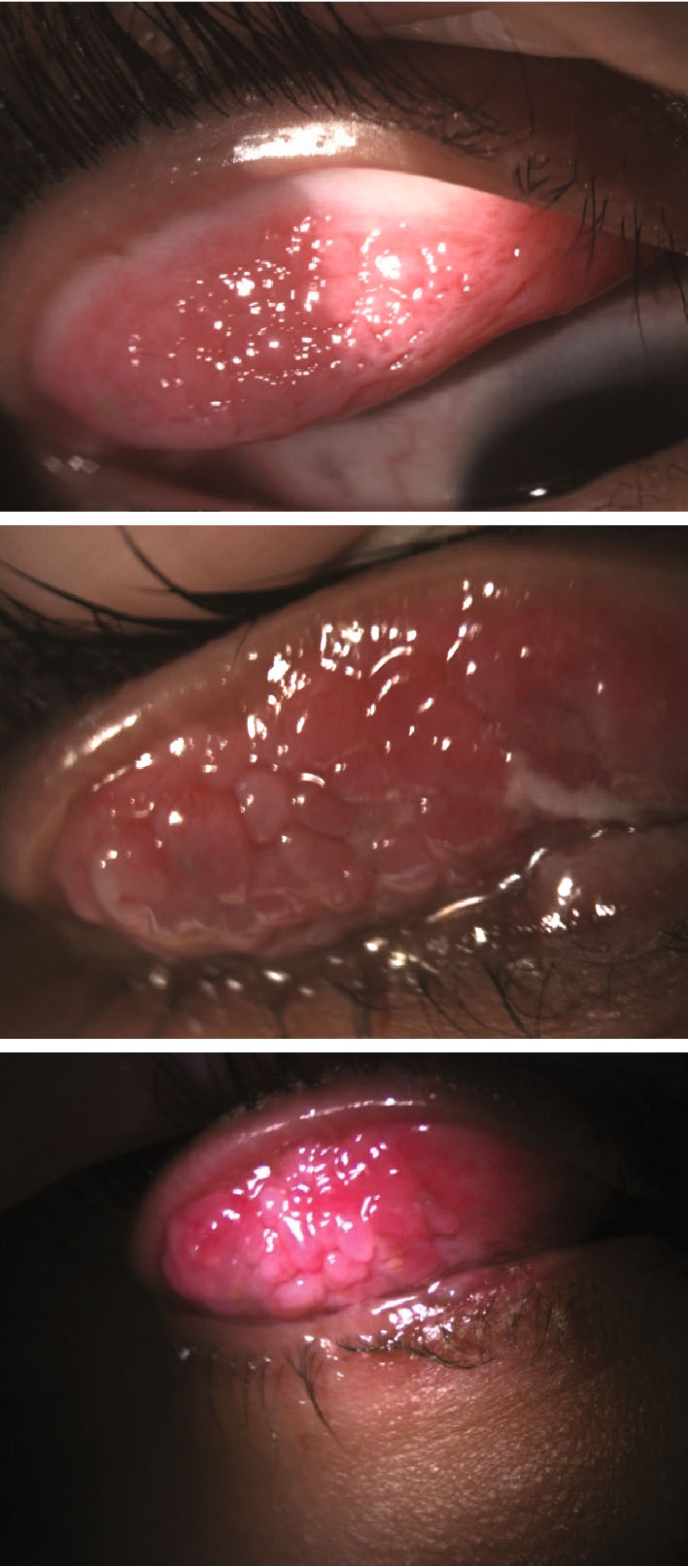
Cobblestones progressivity on superior tarsal of the right eye, on year.

**Figure 5:**
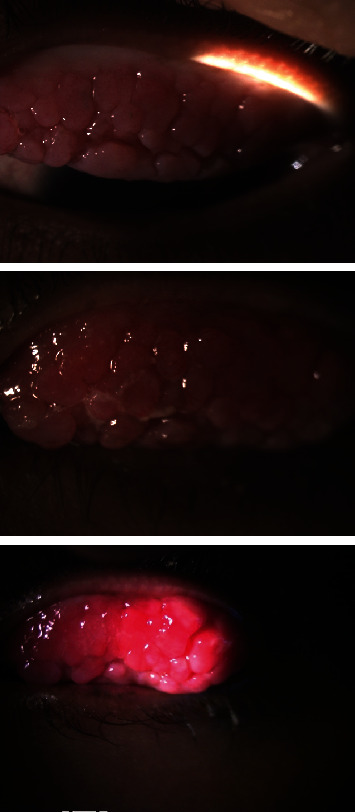
Cobblestones progressivity on superior tarsal of the left eye, on year.

In the second year, 6 months after the second episode, the patient once again complained of itching, watery discharge, and a sensation of foreign body presence in both eyes. Additionally, the patient reported blurred vision and sensitivity to light, particularly in the left eye. Upon examination, it was found that the vision in the left eye had decreased from 1.0 to 0.7. The ophthalmological evaluation revealed the presence of giant papillae (GP) in both eyes, leading to the formation of a shield ulcer in the left eye (Figure 1). Due to the corneal cicatrix formation and decreased vision, it was recommended for the patient to undergo papillary excision and receive a triamcinolone injection. Following the procedure, the symptoms did not recur for 10 months. However, in the third year, the patient experienced a worsening of symptoms with the presence of large cobblestones during the examination (Figure 2). Ropy discharge was prominent, and there was a minor defect on the cornea. Considering the significant cobblestones on the superior eyelids, the decision was made to perform papillectomy, steroid injection in the intratarsal area, and amnion membrane transplantation. The patient's condition remained well-controlled for a year using an antihistamine (Patanol) and artificial tears. After a year, there was a recurrence of symptoms with gradual enlargement of papillae (Figure 3), leading to chronic irritation despite topical antihistamine and steroid. Additionally, the patient exhibited giant cobblestones causing pseudoptosis (Figures 4 and 5), along with an increase in astigmatism in the refractive measurements. In response, a combined papillectomy and autologous conjunctival membrane graft were performed. The results of pathological examination found tissue with nonspecific chronic inflammation with prominent eosinophil.

Following the surgical intervention, the clinical monitoring revealed a relatively smooth upper tarsal conjunctiva throughout the 2-year follow-up period (Figure 5). This resulted in a reduction of mechanical ptosis, the corneal surface in both eyes maintained a relatively clear condition, and GP had not reoccurred. Subsequently, the patient's symptoms were effectively managed through the use of topical medications.

### 2.1. Surgical Technique

**Figure 6:**
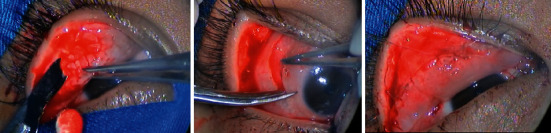
Surgical steps on the right eye; giant papillae were excised, and autologous conjunctival membrane was grafted and sutured directly onto excised tarsus.

**Figure 7:**
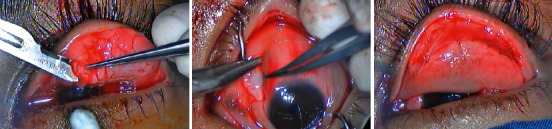
Surgical steps on the left eye; giant papillae were excised, and autologous conjunctival membrane was grafted and sutured directly onto excised tarsus.

The surgical procedure employed in this case was a modified version of the technique described by Nishiwaki-Dantas et al. [9]. The patient underwent general anesthesia for the surgery. To expose the GP, the superior eyelid was everted and secured with sutures. A full-thickness horizontal incision was made approximately 5 mm above the superior border of the tarsus, just posterior to the lid margin, through the conjunctiva. Complete resection of the GP was performed using scissors and a surgical blade. The remaining conjunctival tissue on the tarsus plate was carefully scraped off until a relatively smooth surface was achieved, taking care to avoid the palpebral margin, meibomian glands, and eyelash follicles. Hemostasis was achieved by applying a surgical sponge soaked in diluted topical vasoconstrictor and using bipolar cautery to control bleeding from the tarsal conjunctiva. For the autologous conjunctival membrane graft, subconjunctival injection of bupivacaine was administered to separate the layers between the bulbar conjunctiva. The graft, measuring approximately 5 × 15 mm, was bluntly dissected 5 mm from the limbus and securely attached to the denuded area on the upper tarsus using interrupted sutures of 8-0 polyglactin. Subsequently, a subconjunctival injection of dibekacin (40 mg/mL) and dexamethasone was given, followed by placement of a bandage soft contact lens (Figures 6 and 7).

**Figure 8:**
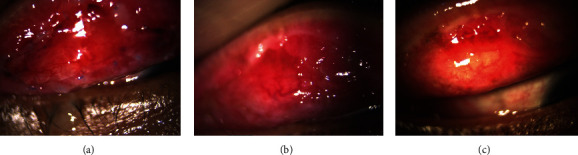
Right eye, post combined papillectomy and autologous conjunctival membrane graft: (a) 10 days; (b) 2 months; (c) 6 months.

**Figure 9:**
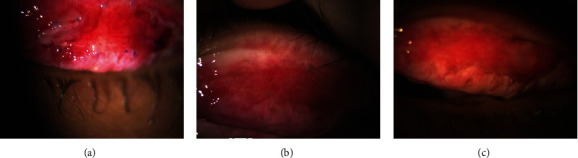
Left eye, post combined papillectomy and conjunctival membrane graft: (a) 10 days; (b) 2 months; (c) 6 months.

Postoperatively, the patient was prescribed topical 1% prednisolone acetate and levofloxacin to be administered every 4 h. Bandage soft contact lenses were worn for a minimum of 7 days to aid in the healing process (Figures 8 and 9).

## 3. Discussion

The management of ocular allergies requires a comprehensive approach, encompassing preventive measures to avoid exposure to specific and nonspecific allergens, along with the administration of topical and/or systemic medications. Mild cases can be effectively treated with cold compresses, preservative-free artificial tears, topical nonsteroidal anti-inflammatory drugs, and topical antihistamines/mast cell stabilizers. In more severe cases, a combination of antihistamines/mast cell stabilizers, immunomodulators, and topical or systemic corticosteroids is employed, either as intensive short-term therapy or as part of long-term treatment regimens. The use of immunomodulators like cyclosporine A and tacrolimus is reserved for cases of steroid-dependent allergic keratoconjunctivitis, especially in situations involving long-term steroid use or contraindications. Surgical interventions, such as resection of GP, may be considered for severe cases resulting in persistent corneal injury [6]. Consequently, for the management of this severe and refractory case, we opted for a steroid injection.

In the treatment of severe acute VKC in children, the supratarsal injection of 20 mg triamcinolone acetonide has demonstrated satisfactory results and good tolerability, providing a safe and viable option for challenging cases. This approach has led to significant improvements in ocular allergy symptoms and signs, resulting in a reduction in the frequency of acute recurrences, although complete disease remission may not be attainable. However, the efficacy of triamcinolone acetate injection on our patient was relatively short-lived [11].

Our patient experienced a recurrence of symptoms within a year, which worsened during examination with the presence of giant cobblestones. Despite ongoing treatment, the patient's symptoms persisted, and the existing GP began affecting the cornea. Surgical intervention may be beneficial for patients with GP and a concomitant shield ulcer that does not respond to conventional therapies [6].

Since this is a pediatric patient, we anticipate the need for ongoing surgeries and recurrent episodes. Although literature suggests that the use of MMC can reduce recurrence [12, 13, 14], we chose not to use it based on our judgment. Instead, we opted for an autologous conjunctival graft rather than AMT, as mentioned in other studies. We aimed to preserve the integrity of the graft and minimize the inflammatory and toxic reactions that MMC could cause, particularly to the cornea [15, 16, 17]. Therefore, we only used a modification of the technique by Nishiwaki-Dantas et al. and found that good results were achievable without MMC [9]. Additionally, due to the frequent recurrence and annual allergic episodes, we believe that the inflammation is influenced by systemic factors [18, 19]. We aim to maintain tissue integrity without using antimetabolites and control the disease with topical and systemic medications.

The ideal surgical approach should aim to permanently remove the GP, alleviating corneal irritation, while preserving eyelid anatomy, conjunctival integrity, which houses important secretory and goblet cells, and the tarsal plate containing meibomian glands responsible for producing the oily tear film layer [20]. After undergoing the combined procedure of papillectomy with AMT, the patient experienced a recurrence of complaints after 1 year. One proposed method for managing recurrent VKC involves performing a single surgical resection along with cryotherapy. However, this approach can lead to uneven conjunctival scarring, resulting in eyelid deformities and irregularities on the superior palpebral conjunctival surface [21]. Another option is utilizing an oral mucous membrane graft, but this carries the risk of creating an additional wound inside the oral cavity [9].

The use of autogenous bulbar conjunctiva, as initially described by Nishiwaki-Dantas et al. in 1996 [9], is a straightforward procedure that can be performed alongside papillectomy. It is important to note that this technique addresses the mechanical irritation caused by GP and improves the ocular surface condition but does not directly impact the immunological process. Therefore, patients still require antiallergic medications. At 6 months postoperatively, the patient exhibited an absence of GP, and the tarsal conjunctiva remained smooth without any disturbances on the ocular surface, resulting in a clear cornea. The patient experienced significant improvement in complaints while continuing to manage VKC with antiallergic medications. After a 2-year follow-up period, the patient demonstrated a clear conjunctiva without any indications of GP recurrence.

## 4. Conclusion

VKC is quite straightforward to diagnose clinically, but its management remains challenging, especially in refractory cases and those compounded with corneal complications [6]. Previous research has demonstrated that patients and parents with severe cases of VKC experience a disruption in their quality of life [22]. Therefore, it underscores the importance of providing appropriate and effective treatment to address the challenges associated with this condition. In light of these findings, our aim is to utilize surgical intervention to address the challenges posed by this severe and refractory VKC case. Rareness of the GP case presentation, along with the influence of tropical weather, presents obstacles in achieving optimal management of VKC cases in Indonesia. By performing papillectomy and layering the excised tarsus with autologous conjunctival membrane graft, we can ensure a smoother tarsal surface, provide corneal protection, and alleviate patient discomfort. This surgical technique yields positive outcomes, is easily performed, does not require a secondary procedure, and reduces the recurrence of GP above the transplanted conjunctiva. Therefore, it is a viable option to consider for patients with severe and refractory VKC cases who have a history of previous surgical procedure failure. Nonetheless, it is important to emphasize that exposure avoidance and allergic control remain crucial aspects of treatment.

## Data Availability

The data used to support the findings of this study are available from the corresponding author upon request.
